# Chitosan-Based Materials Featuring Multiscale Anisotropy for Wider Tissue Engineering Applications

**DOI:** 10.3390/ijms23105336

**Published:** 2022-05-10

**Authors:** George Mihail Vlăsceanu, Mariana Ioniță, Corina Cristiana Popescu, Elena Diana Giol, Irina Ionescu, Andrei-Mihai Dumitrașcu, Mădălina Floarea, Iulian Boerasu, Mădălina Ioana Necolau, Elena Olăreț, Jana Ghițman, Horia Iovu

**Affiliations:** 1 Faculty of Medical Engineering, University Politehnica of Bucharest, 011061 Bucharest, Romania; george.vlasceanu@upb.ro (G.M.V.); corinacristiana23@gmail.com (C.C.P.); 2 Advanced Polymer Materials Group, University Politehnica of Bucharest, 011061 Bucharest, Romania; diana_giol@yahoo.com (E.D.G.); madalina.necolau@upb.ro (M.I.N.); elena.olaret@upb.ro (E.O.); jana.ghitman@upb.ro (J.G.); horia.iovu@upb.ro (H.I.); 3 Cantacuzino National Medico-Military Institute for Research and Development, 050096 Bucharest, Romania; ionescuirina@hotmail.com (I.I.); andrei1mihai.dumitrascu@gmail.com (A.-M.D.); lambrumadalina93@yahoo.com (M.F.); 4 Department of Lasers, National Institute for Lasers, Plasma and Radiation Physics, 077125 Magurele, Romania; iulianboerasu@gmail.com; 5 Academy of Romanian Scientists, 54 Splaiul Independentei, 050094 Bucharest, Romania

**Keywords:** chitosan, EDC-NHS coupling mechanism, fatty acid-grafted chitosan, genipin crosslinking, graphene oxide composite, multiscale anisotropic composite, zero-dimensional coupling

## Abstract

We designed graphene oxide composites with increased morphological and structural variability using fatty acid-coupled polysaccharide co-polymer as the continuous phase. The matrix was synthesized by N, O-acylation of chitosan with palmitic and lauric acid. The obtained co-polymer was crosslinked with genipin and composited with graphene oxide. FTIR spectra highlighted the modification and multi-components interaction. DLS, SEM, and contact angle tests demonstrated that the conjugation of hydrophobic molecules to chitosan increased surface roughness and hydrophilicity, since it triggered a core-shell macromolecular structuration. Nanoindentation revealed a notable durotaxis gradient due to chitosan/fatty acid self-organization and graphene sheet embedment. The composited building blocks with graphene oxide were more stable during in vitro enzymatic degradation tests and swelled less. In vitro viability, cytotoxicity, and inflammatory response tests yielded promising results, and the protein adsorption test demonstrated potential antifouling efficacy. The robust and stable substrates with heterogeneous architecture we developed show promise in biomedical applications.

## 1. Introduction

Advances in biological materials, stem cells as well as growth and differentiation factors have made it possible to build tissues in the lab using an engineered extracellular matrix or scaffolds and physiologically active chemicals that restore, preserve, or improve native tissue functions when impaired. To allow cell adhesion, proliferation, differentiation, and new tissue creation, the scaffold works as an artificial construction that must replicate the chemical composition and physical architecture of natural extracellular matrix [1].

Simple constructions with flat two-dimensional geometries were used in some of the first attempts to create biocompatible scaffolds. In the field of skin engineering, scaffolds seeded with fibroblasts and/or keratinocytes make up the majority of these transplants nowadays. The planar grafts’ basic structure often has a negative impact on their vascularization. As a result, researchers are investigating ways to include more complex geometry into the grafts in order to provide better blood flow to the transplanted area [2].

The geometrical and anatomical structure of a scaffold can be purposefully changed to enhance cell or bioactive substance delivery to the target tissue or organ. Specific scaffolds can also be selected to give the cultured cells the essential signals for proper integration. Needless to say, the paradigm of anisotropy implies specifying the scale, as well as the axis. The chemical bonds in a polymer chain may have an anisotropic angular distribution, those polymer chains may be aligned with the fiber axis, and the polymer chains may be incorporated in a chain-folded lamellar crystal that has a preferred orientation. This form of molecular anisotropy is clearly on a much smaller scale than the anisotropic shapes that could govern the non-chemical cues interacting cells might receive on a superior scale. The nature of the anisotropy is primarily determined by the techniques used to prepare the scaffold.

Chitosan (CHT), the main derivative of the second most abundant natural polysaccharide after cellulose, chitin, is a widely used biocompatible polysaccharide used in dental pulp [3], bone and skin in tissue engineering where it can be employed flexibly [4]. CHT chains (which are hydrophilic) can be modified with hydrophobic substances (such as carboxylic acids, especially fatty acids) to produce amphiphilic products which can self-assemble and create nanoparticles (micelles) [5,6,7]. Although such entities are mainly designed as vehicles for drug or gene delivery, they can be employed as components for the development of 2D or 3D structures with anisotropic architecture for other biomedical applications. After they are coalesced, covalent bonding between the copolymer chains is needed to increase the chemical and morphological integrity of the constructs. Due to its reduced cytotoxicity relative to commonly used cross-linkers (aldehydes, carbodiimides, epoxy compounds), genipin has emerged as a preferred cross-linking agent for primary amine groups, and it has been used to develop hydrogels and scaffolds out of amine-containing polymers such as chitosan, collagen and gelatin [8].

Carbon-based nanomaterials are a great starting point for developing 3D tissue engineering scaffolds [9,10]. Because of their unique electrical conductivity, mechanical stability, chemical composition, biocompatibility, and bioadhesion features, graphene oxide (GO)-based nanomaterials make ideal scaffold materials. By osseointegration, stem cell differentiation into a variety of cells, improved neuronal growth and branching, and increased production of vascular endothelial growth factor (VEGF)/pro-angiogenic gene for cardiac repair, graphene components have dramatically improved the cytocompatibility of biomaterials [11,12,13]. Moreover, polymer matrices supplemented with particular shares of graphenic materials exhibit extraordinary morphologies as GO initiates the formation of rougher topographies and ordered pore frameworks [14,15].

Palmitic and lauric acids, polyunsaturated fatty acids (FA), are low toxicity biodegradable surfactants that can self-assemble into a variety of forms in aqueous solutions and form micelles for various purposes in biomedical research [16,17]. On their own, they easily penetrate the phospholipid bilayer of the cell membrane, disrupting its structure and fluidity, allowing it to be rapidly absorbed by cells. They can also impact the chemosensitivity of tumor cells [18,19,20]. Because of chemical alterations, these self-assembled structures can respond to stimuli such as pH and temperature. These characteristics make them molecules of particular importance for tuning macroscopic properties. Fatty acids become less water soluble as chain length increases. In our study, lauric acid (12-carbon atom chain) and palmitic acid (16-carbon atom backbone) were used due to their different lengths, which were anticipated to induce a strongly packed structuration within the copolymer as well as uneven interaction with the polysaccharide and interesting templates on the molecular level.

As a result, these FAs were chosen as the hydrophobic component to modify chitosan in our study, and an amphiphilic FA-g-chitosan (CHT-g-FA) copolymer was devised and synthesized with the goal of improving chitosan morpho-structural features as required in the field of tissue engineering (TE). These materials were made by combining N, O-acylation with a carbodiimide coupling agent in an acidic environment, resulting in partial protonation of amino groups and the reaction of the less reactive primary hydroxyl groups of chitosan with fatty acids. The partial protonation implies that not all the primary amines were transformed into secondary amines, granting the option of performing additional reactions to the retained functionalities.

Hydrogel-oriented scaffolds have a wide range of applications enabled by 3D structure and tunable chemistry fit for modelling specific topographies or achieving irregular shapes, either by controlling their gelation and flow kinetics in situ (injectable hydrogels [21]) or by employing revolutionary means of fabrication such as 3D printing [22,23] and in-depth/multi-axis durotactic gradients by altering the number of crosslinks between polymers [24]. Recent research in the field has demonstrated that adjusting the mechanical characteristics of the hydrogel changed the attachment rate of chondrocytes and fibroblasts implanted on the hydrogel in animal models [24,25].

This work, therefore, describes the manufacture of all-natural film-like structures consisting of CHG-g-FA as a starting point for designing architectures with multiscale anisotropies. Next, CHT-g-FA was crosslinked with Gp and reinforced with GO, the most promising carbonaceous nanomaterial for TE prospects, closely monitoring particular physicochemical development by Fourier Transform Infrared Spectrometry, Dynamic Light Scattering, and Scanning Electron Microscopy, as well as aqueous media swelling and stability and in vitro outcomes of the systems against the L929 cell line.

It is, to the best of the authors’ knowledge, one of the pioneering works on developing cell supports from polymer vesicles as fundamental units and the first consisting of chitosan-fatty acids core shells (micelle-like bulk assemblies). Fatty acid grafted chitosan and other polysaccharide chains have been extensively employed in the development of drug and gene delivery systems [26,27,28]. However, copolymer structuration shows promise in scaffold development due to its morphology and chemical patterning. The cvasi-spherical morphology of the initial self-packed copolymer fusion constructs provided first base support for achieving irregular surface topography and a variable micromechanical profile uncharacteristic of pristine biopolymer films. The experimental design also tested the use of an emergent non-toxic crosslinker and of GO for improved cellular interaction, enhanced contact area surface and assorted chemical and micromechanical domains. The polyphasic formulations could become one of the first examples of the new generation of biomaterials featuring intrinsic anisotropy, easing the fabrication of devices with multi-scale variability.

## 2. Results and Discussion

Anisotropic substrates and coatings per se designed for medical devices are appealing choices for regulating the materials’ interactions with cellular bodies, directing the lineages response and amending the outturn of integration. We compare and discuss both microscopic and mesoscopic anisotropy influences, such as the topography and molecular level interactions, similar to those that may occur in a molecularly patterned organization with preferred chain orientation that was induced during the processing procedures used to prepare the scaffold.

### 2.1. Fourier-Transform Infrared Spectroscopy (FTIR)

**Figure 1:**
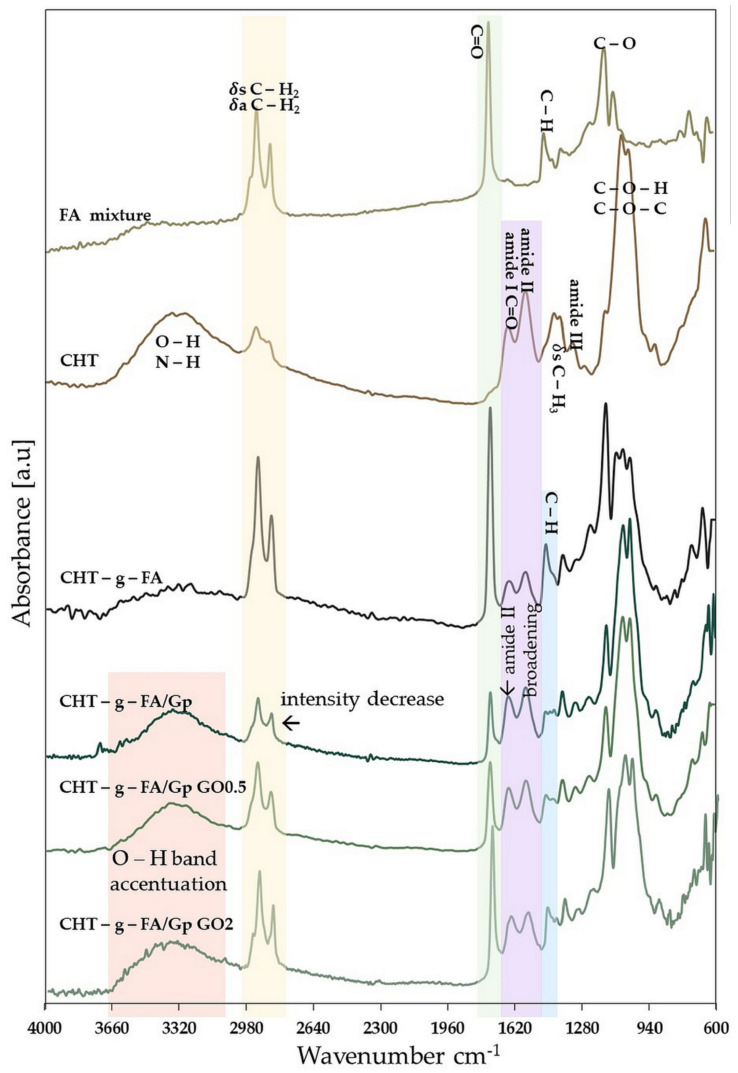
FTIR spectra of precursors and CHT-g-FA copolymers and composites.

FTIR experiments were performed to investigate the successful chemical modification of chitosan with palmitic and lauric acids and also structural changes that emerged after Gp crosslinking and GO addition. Figure 1 shows absorption bands for the FTIR spectra of FA, CHT, CHT-g-FA and its derivatives.

Despite a strong signal (1552 cm^−1^) emerging in the amide II peak range, there were absorption peaks at 1652 cm^−1^ and 1313 cm^−1^ that are indicative of chitosan and have been described as amide I/C=O vibration of the acetyl groups and amide III peaks [29]. The CH_3_ symmetrical deformation mode was indicated by the adjoining peaks at 1380 cm^−1^ and 1406 cm^−1^. The amine N–H symmetrical vibration blends with the OH absorption band, which can be used in conjunction with 1652 cm^−1^ for the appraisal of chitosan deacetylation [30]. The usual C–H stretch vibrations have peaks at 2856 cm^−1^ and 2925 cm^−1^ [31].

The absorption bands in the fingerprint region below 1100 cm^−1^ show numerous modes linked to carbohydrate structure, such as C–H deformation, C–O or C–C stretching; the spectrum’s very strong peaks at 1033 cm^−1^ and 1066 cm^−1^ also reveal the C–O (C–OH and C–O–C in pyranose rings) bending in chitosan. The carbohydrate’s distinctive absorption corresponds to the secondary peaks at 642–900 cm^−1^ (CH bending out of the plane of the ring) [32].

Regarding the spectra of the FA mix, strong signals are accentuated at 2923, 2850 (oil chain asymmetrical and symmetrical C-H vibration), 1745 (originating from the carbonyl (C=O) stretching), 1461 (C–H in CH_2_ bending), 1151 and 1112 (C–O stretching) cm^−1^. Additionally, –HC–CH– cis vibrational and CH_2_ wagging (CH_2_)_n_ rocking out of plane signals amass in the range of 968–632 cm^−1^ [33].

After the coupling reaction, the CHT-g-FA hybrid spectrum exhibits slight changes but perpetuates intense signals associated with the oil component at 2800–2900 cm^−1^, 1745 and 1159 (redshifted) cm^−1^. Compared to the spectrum of the original chitosan, the signals from 1550 cm^−1^ and 1641 cm^−1^, which are specific to the primary amine and the acetyl group, decrease in intensity. In the case of the primary amine, this is due to its transformation into a secondary amide as a result of the coupling reaction, a process that also results in a shift of the signal to 1560 cm^−1^ as a result of the dimming of the amide group. The specific polysaccharide spectrum exhibits a decrease of the 1652 cm^−1^ acetyl group that is consistent with the water affinity of the final product independent to the pH value [34]. A new peak at 1458 cm^−1^ attributed to C–H vibration of the methyl groups appears. However, the double split signal around 1050 cm^−1^ from the precursor biopolymer exhibits three independent equal peaks as a 1103 cm^−1^ signal arises in the region, associated with C–O stretching [35].

After genipin crosslinking, a more distinct structuration of the material manifests. The oil-phase specific peaks decrease drastically as Gp bonds with the remaining amine groups from the chitosan chain and favors the hydrophobic/hydrophilic domains separation. Between 1000 cm^−1^ and 1100 cm^−1^, C–N stretching vibrations, and C–C–N bending vibrations are underlined. Absorptions from C-N stretching vibrations and C–OH stretching vibrations, which are more common after crosslinking with genipin, can be linked to the increase in peaks in the range extending up to 1400 cm^−1^. Moreover, the amide II band at 1546 cm^−1^, characteristic of N–H deformation, tends to broaden after crosslinking [36]. The OH band is more pronounced in this composition. GO compositing (with both ratios) does not cause major changes, apart from the accentuation of the 3000 cm^−1^ and 3600 cm^−1^ broad band, due to the high amount of hydrophilic moieties specific to this carbon species [37]. This behavior, that we only address in a qualitative manner, can be due to GO agglomerate formation within the composite systems.

### 2.2. Dynamic Light Scattering (DLS) Measurement

The many interactions that might occur between the components of the systems are among the essential elements that drive the integrity, stability, and overall performance of the planned anisotropic scaffolds as potent biomaterials [38]. Table 1 shows the hydrodynamic parameters (hydrodynamic size—d, polydispersityindex—PdI, zeta potential—ζ, diffusivity—D) that were gathered by means of Dynamic Light Scattering measurement.

Interestingly, the carboxylic and hydroxylic functionalities, attributed to the negative electrokinetic potential of polysaccharide, graphene oxide moieties and electronegative fatty acids do not alter the charge of the systems, probably due to the fact that upon grafting, the amphiphilic hybrid gathers a conformational geometry whereby the positively charged groups are oriented towards the exterior and the negative domains are recruited inwardly.

There is a significant difference in the hydrodynamic characteristics of the initial modified CHT with respect to precursor and processed CHT-g-FA, which is primarily due to their chemistry. The corresponding sphere diameter drawn around the studied molecules with the same diffusion behavior is referred to as the species’ mean hydrodynamic size (d, nm). The electrical properties of an entity are denoted by the zeta potential (ζ, mV), which is an indicator of surface charges and an appropriate index of colloidal species interaction magnitude [39]. CHT’s positive zeta potential is due to the intrinsic cationic feature (its core backbone is comprised of a primary amino group that creates a positive charge in biological fluids) of the polysaccharide that preserves after FA grafting and compositing. To begin with, the CHT-g-FA suffered a strong size decrease in comparison with CHT (536 nm/751 nm) that could be attributed to the distinct molecular orientations of the FA domains. The carbohydrate chains might tend to form clusters which could mold the geometry of the CHT backbone into a coating-like formation. This type of assembly would generate more compact molecular constructs, with a more uniform polydispersity (PdI = 0.488), as indicated by the measured values tabulated above. In the presence of diverse molecules/media, similar behavior of natural polymers modified with oil chains has been frequently observed in the literature [40,41,42].

**Figure 2:**
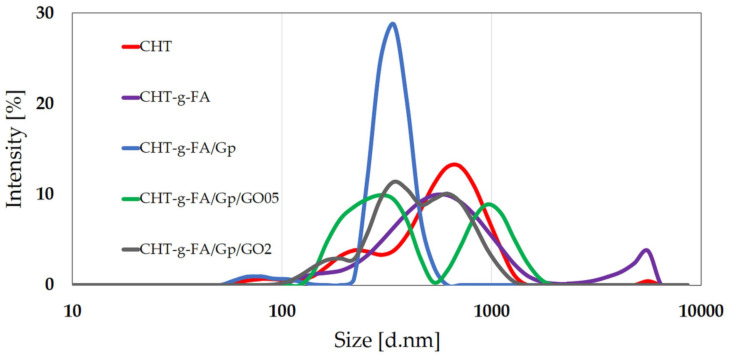
Size distribution chart of CHT and modified CHT constructs from the DLS technique.

According to the results, the Gp addition seems to strongly impact the hydrodynamic feature of the FA grafted systems. Gp is able to covalently bond the CHT-g-FA construct through CHT functionalities, leading to agglomerates with increased mean diameter and more narrow bimodal distribution compared with the multimodal broad size distribution of the CHT-g-FA population (Figure 2). Apparently, Gp addition seems to be the only factor that impacts this feature of the FA grafted systems. Gp is able to bind CHT chains and also the CHT-g-FA construct, as the hydrodynamic diameter reaches maximum values within the batch. Additionally, Gp induces the highest zeta potential (+51.4 mV) by targeting the residual primary amines available after copolymerization.

The outcome of compositing with GO seems to be related to homogeneity disruptions on a molecular patterning level that could lower the system’s hydrodynamic isotropy. In the case of multicomponent systems (CHT-g-FA/Gp/GO05, CHT-g-FA/Gp/GO2), it appears that besides genipin-mediated interactions, a combination of processes, such as hydrophobic interactions, dispersion forces (van der Waals), and hydrogen bonding effects cause conformal modification, influencing the diffusion coefficients and collective hydrodynamic features. The velocity of a colloid is directly related to its diffusion coefficient, which is inversely proportional to its diameter and directly proportional to its mobility. As the size of the agglomerates grows larger, the individual or collective diffusivity parameter decreases. The decrease by almost half in the electrokinetic potential of GO composites (ζ = +33.4 mV) when compared with CHT-g-FA/Gp (ζ = +51.4 mV) indicates the presence of multiple H-bonds between modified polysaccharide and graphene oxide functions, primarily COOH. The CHT-g-FA/Gp/GO05 system presents the lowest diffusivity (D = 0.645 μm^2^ s^−1^) with a bimodal broad size distribution. The CHT-g-FA/Gp/GO2 system is characterized by superior diffusivity (D = 0.759 μm^2^ s^−1^), lower hydrodynamic size and a more uniform colloidal ensemble (PdI = 0.572), as can be seen in Figure 2. However, the stability of the component is not increased, indicating that GO might locally stack due to specific pi-pi interactions, stronger than the ones formed with the matrix. These results are in agreement with other studies that address the behavior of polysaccharide chains in the presence of graphene materials [43].

### 2.3. Contact Angle (CA) Measurements

Contact angle measurements were used to determine the hydrophilicity and the surface free energy of the samples [44].

**Figure 3:**
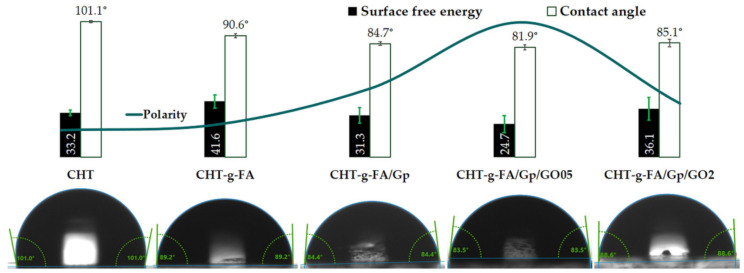
Material polarity, surface free energy and contact angle depictions according to sessile drop measurements.

After copolymerization, the wettability of CHT-g-FA was found to be higher than that of the unmodified CHT due to the preferential assembly of the hydrophilic and hydrophobic domains. Augmented separation of these sectors occurred after crosslinking since Gp brought to surface more hydrophilic moieties. Nonetheless, 0.5 wt.% additivation with GO induced the highest hydrophilicity, whereas the highest GO content contributed to a moderate enhancement of hydrophobic character in the respective composites, probably due to the topographical aspects dictated by the ratio of carbon sheets. Polarity values measured and depicted as a curve in Figure 3 linearly concur with the contact angle values, as the material with the lowest contact angle (CHT-g-FA/Gp/GO05) featured the most enhanced polar trait.

In the present case, SFE generally followed an inverse trend with respect to surface wettability which is rather uncommon. When exposed to air conditions, most solids with high surface energy will lose their high-energy surface as hydrocarbon impurities in the air will adsorb onto the surface of the solid, lowering the surface energy. A surface with a low SFE will generally induce poor wetting and, as a result, a high contact angle. This is because the surface is incapable of forging strong bonds; therefore, breaking bulk bonding in favor of engaging with the surface offers less energy return. CHT-g-FA features the highest SFE value as the reactive hydrophilic functionalities are unrestrained. An important decrease is measured after crosslinking, a procedure that binds the reactive moieties of the copolymer chains. The most relevant drop occurs though after the addition of GO since the additional interactions that form between the continuous and dispersed phase increase the stability of the composite. The reactivity and stability of the composition are in agreement with the degradation behavior monitored in the batch for up to two months.

### 2.4. Scanning Electron Microscopy (SEM)

**Figure 4:**
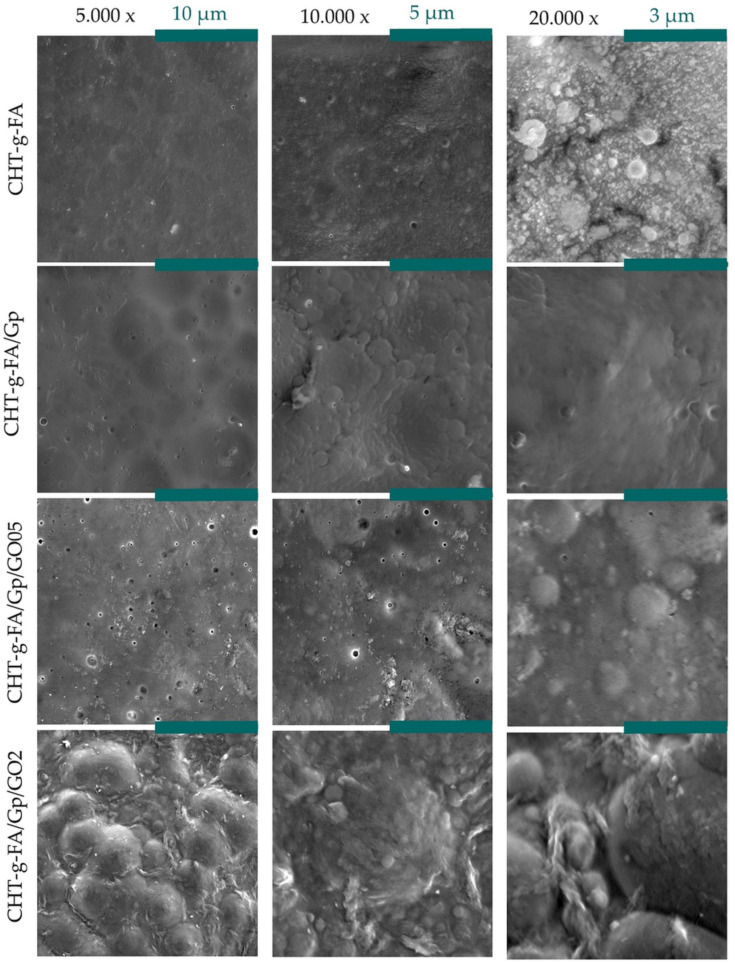
Surface morphology of the synthesized materials captured by means of SEM.

The films that resulted show an uncommon surface morphology when compared with other polymer films. Typically, such devices show smooth surfaces with few particularities, especially when observed by SEM. However, the present batch is characterized by irregular surface features. In Figure 4, at higher magnification (20,000×), CHT-g-FA shows conjoined spheroid-like entities that render a sinuous surface that can potentially support cell attachment. DLS analysis confirmed that upon coupling, the hybrid material tends to have a micelle-like structuration whereby the hydrophobic chains of FA are recruited inside. The irregular size particles adhere, but the interactions between them are insufficient to enable their fusion into an even structure. This impediment might be due to the fact that the electrokinetic potential of new polysaccharide is highly complex with opposing charges that have the potential to govern the assembly in film structure and generate anisotropic patterning on a supra-molecular level.

CHT-g-FA/Gp, in contrast, is smoother, due to the fact that genipin binds macromolecular chains, induces a stress in the organic matrix and amplifies the tension in the network. This levelness of the crosslinked film enables the observation of pocket-like unit formations and the fashion in which they bind.

Nonetheless, alongside GO addition, this type of organization of the fatty acid grafted polysaccharide changes and the spheroids becomes more apparent; moreover, the roughness increases radically, not only due to the projection of the dome-like formations. Particular features appear in the case of the GO composites that might be phase separations of poorly modified chitosan chains, which are more prone to mingle with graphene oxide particles. These types of structuration are more visible in the case of CHT-g-FA/Gp/GO2 where the curvaceous surface is sprung with irregular shaped deposits of polymer/polymer coated GO.

All things considered, GO addition is clearly influencing the morphology of the films to form highly rugged architectures. The anisotropic nature of the films stems firstly from the ordered domains structuring based on the local charges in the copolymer, which can be seen as the fundamental patterning unit of this batch. Building on this, Gp and GO additions generate cascading shifts with enhanced heterogeneity. As a result, the morphology of the material becomes more complex on a multiscale, thus proving better lodging for cell cultures. Additional insights on the superficial irregularities of the samples can be found in the Supplementary Materials.

### 2.5. Nanoindentation

Because nanomechanical sensing has an impact on future cell behavior [45], it was of interest to screen how the FA copolymerization, crosslinking and presence of GO affects the mechanical properties at the surface. Instrumented indentation tests allow the determination of mechanical features at the surface as they are able to discriminate differences induced by low concentrations of nanospecies [46,47,48] or changes induced by a physical crosslinker [48].

**Figure 5:**
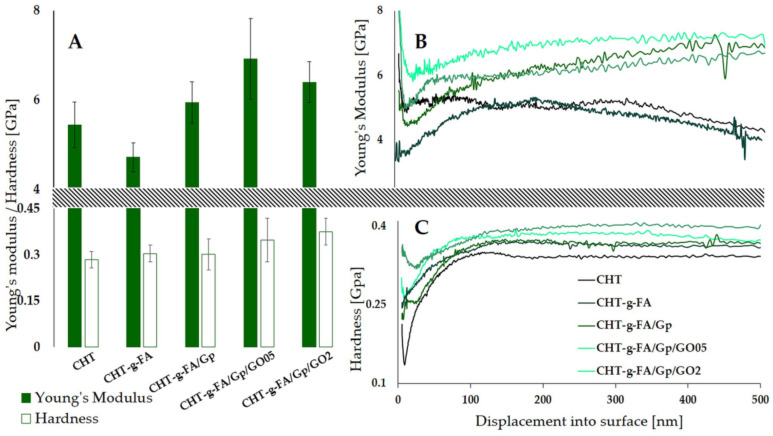
Influence of copolymerization, crosslinking and GO compositing on the nanomechanical properties of CHT formulations up to a maximum depth of 500 nm: (**A**) representative Young’s modulus and hardness values at 100 nm displacement into surface; (**B**) Young’s modulus as a function of the indentation depth; (**C**) hardness as a function of the indentation depth.

Figure 5B,C shows the E and H variations as a function of penetration depth, as well as the averaged values of 10 measurements recorded between 90 and 100 nm indentation depth (Figure 5A) for all tested compositions. Hardness progressively increases with the addition of the crosslinker and GO embedding since they, on the one hand, compact the polymer network and, on the other, act as harder centers across the continuous phase. In addition, the elasticity decreases after crosslinking and compositing, most prominently for the 0.5 wt.% GO formulation, probably since the carbon nanoparticles had been better dispersed and interact stronger with the matrix, in contrast to CHT-g-FA/Gp/GO2. The recorded E values for pristine CHT-g-FA range lower those for the initial polysaccharide material suggesting a softener effect of FA onto the CHT. However, both the crosslinking and GO filling lead to a reinforcing effect on CHT-g-FA matrix with a maximum E reached for CHT-g-FA/Gp/GO05 (6.92 GPa), thus confirming the firmest compositing formula and the ability of GO at low ratios to promote better mechanical support. These results are in agreement with [49] which reported that the addition of small amounts of graphene (0.1–0.3 wt.%) indeed exhibit an influence over elastic modulus of chitosan films. Increasing the GO content from 0.5% to 2% enhances the contrast amongst the constituent phases, as reflected in the E. The lower E value obtained for the composition with a higher amount of GO could be explained by a different organization of GO sheets into the polymeric matrix, probably an out-of-plane direction of GO which causes an unbalanced stress at the GO-polymer interface and leads to weaker mechanical properties when compared with CHT-g-FA/Gp/GO05 where GO is more homogenously distributed within the polymeric matrix [49]. This observation is supported by the SEM images (Figure 4) which show a better dispersion of GO sheets into the polymer matrix for the ratio of 0.5%.

### 2.6. Aqueous and Enzymatic Degradation

**Figure 6:**
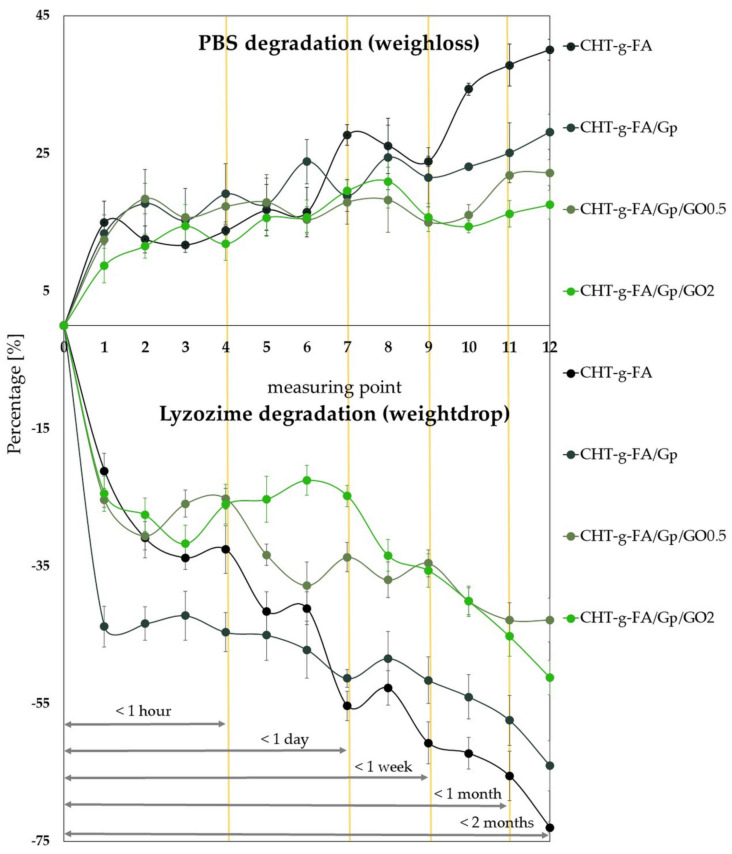
Fused depiction of the specimen weight loss after degradation in PBS and in specialized enzyme rich environment, as a function of temporal milestones (minutes, hours, days, months). For better visualization, one profile was outlined in positive values lost after incubation, while the other one was outlined in negative variation.

The degradation of the 4 compositions batch was studied from two perspectives: in PBS and under enzymatic attack (chitosan specific lysozyme). For both media, we pinpointed multiple time milestones based on the 12 measuring points we fixed (Figure 6). In both cases, significant weight loss was recorded in the first minutes (<1 h) after immersion, since the repulsive electrostatic interactions were probably boosted in aqueous media and expelled the less embodied domains from the film volume. The trend continued significantly decelerated up to 24 h and in the case of the samples degraded in PBS, rather insignificant changes occurred for the batch, with the exception of CHT-g-FA.

Important variations were observed in the case of enzyme degradation. Up to 24 h, the CHT-g-FA and the Gp crosslinked material suffered a substantial decrease in mass, a trend that continued at a slower pace for the following intervals (<7 days, <1 month, <2 months). In contrast, the degradation of the GO composites in lysozyme followed a less steep and more constant slope, becoming slower from the hour range to the months outcomes. Furthermore, after two months, these two compositions were most stable, provided that GO impeded enzyme penetration.

Regardless of the degradation environment, the CHT-g-FA suffered the highest degradation while GO addition provided additional stability to the aggressive media. Moreover, Gp crosslinking significantly enhanced the preservation of the material up to two months. As a general trend, the lysozyme mediated degradation was more efficient since the weight loss registered was generally twice as substantial.

### 2.7. Swelling Degree

In this study, the network features an oscillatory swelling behavior, the sinusoidal-like profile being attributable to the heterogeneous assembly of hydrophilic and hydrophobic domains. Because the side chain of the polysaccharide confers the hydrophilic character, and upon copolymerization the material aggregates in core-shell-like assemblies, swelling tends to occur in bursts and contractions that expel some of the absorbed volume. This phenomenon is more pronounced at the beginning, until the entire specimen is soaked; as more and more of the disparate hydrophobic phases get wet, the soaking media encounters the inner hydrophobic aggregates which repulse water.

A relative equilibrium was achieved after 72 h even though variations still emerged, suggesting that the distinct building blocks of the structure feature different dimensions and degrees of accessibility for a penetrating soaking agent; this proposed kinetic mechanism is supported by the multi-scale structuration of particles unveiled by DLS measurements (Figure 2). Hence, this high heterogeneity derived from the size distribution of the copolymer unit assemblies, appears to form a dense coalescence that obstructs swellability. For prospective applications in vivo, this restrained process can be valuable since the materials would display a lean integration within the host, with negligible morphological variations and minimal stress onto the adjacent tissues.

The establishment of a well-defined core–shell structure in aggregates, in which the majority of grafted molecules are concentrated in cores and the largest share of hydrophilic groups form the shell, is thought to be connected to their stability in simulated physiological environments. Because not all units were protonated under the experimental conditions, charged groups may transfer from the aggregate’s core to the shell, stabilizing the system further. Because of the tiny amount of charged groups in shells and steric hindrances caused by hydrophilic chain fragments (loops, free ends, etc.) concentrated on the surface, these aggregates stabilize. The role of steric stabilization becomes more prominent as the degree of charge of macromolecules decreases. One of the reasons for solubility/dispersibility of hydrophobically modified chitosan in neutral environments is the inhibition of chitosan’s inclination to crystallize after the introduction of fatty acids side substituents. As a result, the aggregates of hydrophobically modified chitosan become compact and stable even in neutral solutions as the pH rises. This circumstance is critical for broader applicability in medicine.

**Figure 7:**
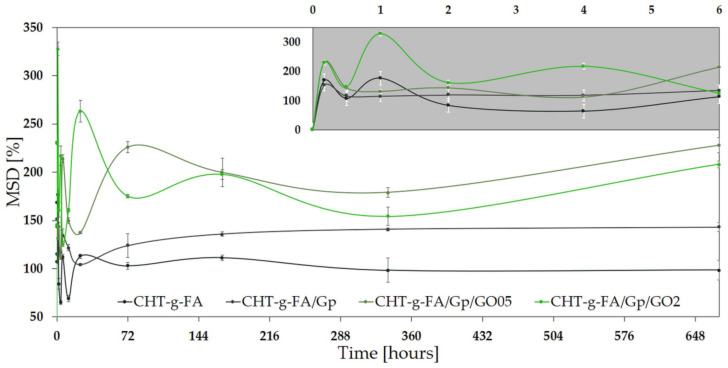
Maximum swelling degree achieved up to 672 h immersion in PBS. The inserts depict the variation from 0 to 6 h.

In physiological buffer (PBS) media, the hydrophilic character and swelling properties of the newly synthesized materials were measured after various incubation times (up to 4 weeks) as maximum swelling degree (MSD %) in Figure 7 (at neutral pH of 7.4). The time is expressed in hours and for short intervals, Figure 7 insert renders more clearly the oscillatory behavior upon aqueous solution contact.

CHT is a compound with a high affinity for water; however, upon copolymerization, we described an increase in hydrophilicity in the chase of the CHT-g-FA batch, a differentiating property that should have a significant impact on the system’s swelling behavior. Furthermore, GO supplementation was described as a favorable filling agent that significantly enhances the capacity to bind large volumes of water in polymer composites. The swelling capacity of the composites generated by interaction with GO revealed significant rise in MSD (values in the range of 200–230%).

Interestingly, the swelling capacity of the modified chitosan materials is significantly lower compared with the behavior biopolymer-based composite materials exhibit generally [50]. A link between the crosslinking event and the degree of swelling in aqueous media under neutral conditions may be defined, according to Figure 7. Overall, the MSD rapidly (in 10 min) reaches values of more than 100% but is relatively constant over longer time intervals (weeks), gradually exceeding 200% for the GO composites (228% and 208%). Such profiles might favor the use of this type of composite in coating applications for implantable metallic devices for orthopaedics.

It was shown that the Gp crosslinking process has a significant impact on the material’s absorption capacity in PBS: genipin crosslinked networks acquired larger swelling than uncrosslinked networks. By the end of measurement, CHT-g-FA/Gp absorbed up to 45.1% more water than CHT-g-FA. After GO addition in the Gp containing formulation, the tendency was only increased, but at a slow rate; the 0.5 wt.% GO containing network was able to accommodate 59.4% more PBS, whereas the CHT-g-FA/Gp/GO2 accommodated 45.6% extra. The nature of H bonding connections, which would readily interact with water, causes more swelling in GO containing crosslinked networks. After crosslinking and at increasing GO concentrations, the development of H-bond interactions is favored, resulting in dense network architectures that delays swelling equilibrium but enables the absorption of larger amounts of water and the results in large swelling extents. These characteristics could favor the nutrient exchange within the matrix, essential to the support of implant integration.

In terms of chain extension, the crosslinking pattern of genipin appears to be more permitting to water permeation and housing; even though genipin immobilizes the polysaccharide chains, upon bonding, intermolecular spaces are formed among the hydrophilic sectors which can provide significant volumes for PBS absorption.

Swelling studies in PBS revealed a larger MSD for the composites, confirming the contact angle measurement and the fact that polarity promotes more network relaxation in aqueous solution and so increases swelling. Furthermore, the morphological characteristics of the constituent copolymers and fillers have an impact on water infiltration.

While the swelling of CHT-g-FA specimens is mainly influenced by processes at the interface with the surrounding environment, fluid absorption is also influenced by the phase alternation within the materials. As a result of the substantial interparticulate gaps in the GO-containing samples, water permeability is expected to be increased. Our findings further imply that increasing the heterogeneity in the composite networks increases the hydrophilic character of multi-component systems.

Swelling degree and comparative degradation testing revealed that the formulations fluctuate less with the addition of GO, suggesting that apart from potential use in scaffold manufacturing, CHT-g-FA compositions could potentially be employed in various types of coatings (catheters, metallic devices) due to their durability and moderate swelling in aqueous media.

### 2.8. Biological Assessment

**Figure 8:**
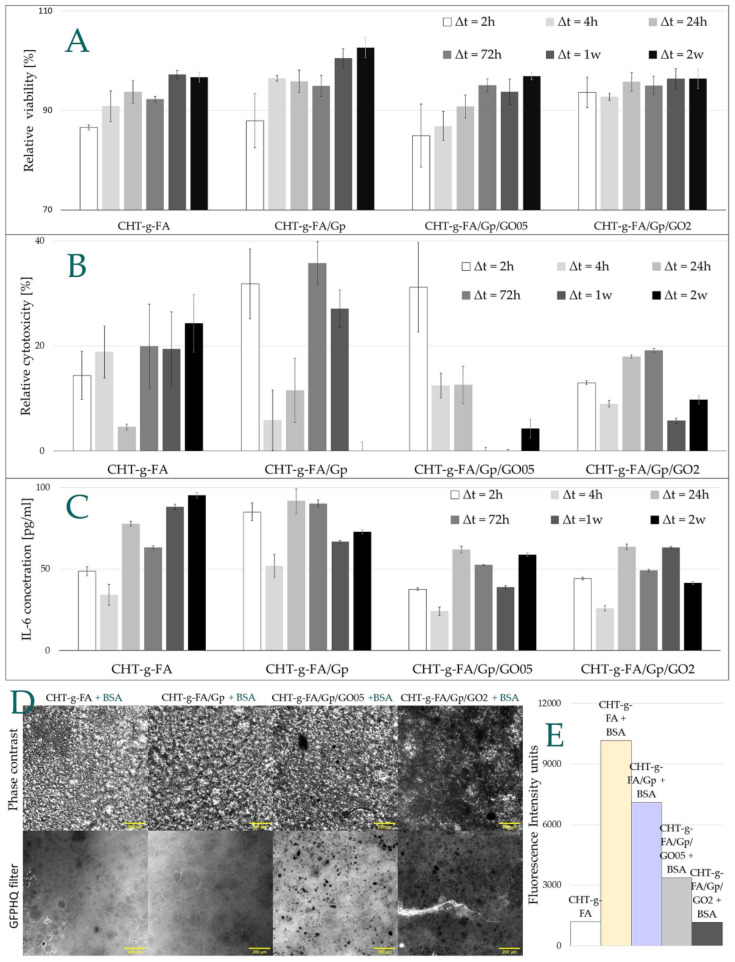
(**A**) Relative viability according to MTT measurements; (**B**) relative cytotoxicity calculated from LDH investigation; (**C**) IL-6 expression measured by ELISA assay; (**D**) microscopy images of chitosan-FA copolymer after incubation in BSA solution whereby the scale bar envelops 200 µm; (**E**) fluorescence intensity of FITC-modified BSA adsorbed onto the chitosan derivatives surfaces. measurements due to its intrinsic quenching ability, as the fluorescence is diminished with the GO ratio to copolymer increase. Still, this composite copolymer can be a good candidate for applications where antifouling properties are required.

In order to address the potential use of the synthesized chitosan derivatives in biomedical applications, preliminary in vitro studies were carried out. They were designed to offer primary indications with respect to the cytotoxicity induced by the composites, cell viability and inflammatory response associated with them (Figure 8). The statistical analysis can be consulted in the Supplementary Materials. In addition, protein adsorption was investigated by fluorescence microscopy against a FITC-modified BSA protein model (Figure 8).

Standard 3-[4,5-dimethylthiazole-2-yl]-2,5-diphenyltetrazolium bromide (MTT) and lactate dehydrogenase (LDH) assays were used to evaluate the relative cell viability and cytotoxicity induced by CHT-g-FA control and derivatives using an indirect extraction method that investigates both the cytotoxicity given by a material and the toxicity of any possible leaching (unreacted) reagents, solvents or by-products from that material. As such, the guidelines of the ISO standard 10993-5 were followed and the investigated materials were incubated in simulated physiological conditions (cell medium, DMEM, at 37 C) and at predefined time points part of the medium was collected to assess the cytotoxicity. Time points from 2 h up to 2 weeks were used. The obtained data are summarized in Figure 8. The cytotoxicity was calculated by incubating healthy L929fibroblast cells in the collected supernatants and measuring the relative cell viability after 24 h. Obtained values were normalized against healthy cells cultivated in fresh DMEM cell culture medium, with a set viability of 100%.

A relative cell viability above 85% was found for all materials, well above the threshold of 70% recommended by the ISO standard, with one case even showing an enhanced cell proliferation behavior, i.e., CHT-g-FA/GP-timepoint 2 weeks. There was a general trend for all materials to better support cell viability in time, probably due to the fact that the solid support reaches equilibrium in liquid media after an extended incubation time in relationship with the swelling behavior as previously discussed. Additionally, genipin crosslinked materials were observed to have behavior superior to that of the control, and GO composites seemed to manifest the least variable behavior in time.

When considering the GO grafted materials, an apparent improvement in cytotoxicity, constant over time, was seen for the CHT-g-FA/Gp/GO2 compared with CHT-g-FA-/GO0.5. The latter presented the lowest relative cell viability for t = 2 h, associated also with a significant standard deviation value, most likely due to some release/free floating component such as GO within the composition. The LDH assay data supports our theory, showing the highest values after 2 h for the CHT-g-FA/GP/GO derivatives, with a significant difference between 0.5% and 2% loads.

Apart from the immune and non-immune cells, some cytokines mediate post-implant inflammation and it is of interest to evaluate their expression. Immune system events as well as inflammatory responses or bone metabolism are affected by IL-6 which is involved in chronic inflammation (autoimmune diseases) and acute inflammation during the initial immune response and wound healing [51].

IL-6 expression was measured for the modified chitosan derivatives to assess the possible occurrence of inflammation post implantation. The variation of IL-6 expression was monitored in time and the results are depicted in Figure 8C.

The clinical implications of IL-6 expression are multiple and should consider the prospective application; generally, values of up to 20 pg/mL can be measured in samples harvested from healthy individuals as IL-6 can be involved in various metabolic processes. However, elevated serum IL-6 concentrations up to almost 1 ng/mL can be detected in patients who have undergone surgical interventions [32]. In the present study, the investigated chitosan materials induced IL6 values between two- and four-fold higher than general values. This can be expected post an initial 2 h incubation of the chitosan derivatives in the culture medium, as most of the possible cytotoxic, mutagenic or allergic components trapped within the material, or the material itself will affect the organism. Important is that this effect will diminish rapidly and will allow for a normal regulation. In this respect, a 25% decrease in IL6 pro-inflammatory marker expression can be seen after 4 h incubation, followed by a sharp increase after 24 h. A clear differentiation between FA grafted chitosan mixtures, crosslinked or not with genipin, versus GO loaded chitosan materials was seen. Thus, post 24 h incubation, IL6 expressions of 60–90 pg/mL for CHT-g-FA and CHT-g-FA/Gp were observed compared with 40–60 pg/mL values for CHT-g-FA/Gp/GO, indicating a surprising decrease of the inflammatory response in the presence of GO, which was below the lower thresholds reported in literature [52,53].

BSA adsorption on the four chitosan derivative surfaces was investigated via fluorescence microscopy (Figure 8D). Phase contrast and fluorescent images were taken for the CHT-g-FA reference sample (denoted CHT-g-FA + BSA) and for the derivatives compositions post an overnight incubation (disc-shaped specimens) in a 1 mg/mL BSA solution, fluorescently labelled with a FITC moiety.

As was expected, a significant amount of BSA adhered on the surface of modified chitosan surfaces. A negative dependence between the protein adsorption amount and the graphene content was quantified in empirical fluorescence intensity units (Figure 8E). As such, a maximum amount of protein was adsorbed on the modified chitosan sample, and decreases with genipin crosslinking and the amount of graphene incorporated within the biomaterial composition. It was interesting to monitor this unexpected behavior that can be associated partly with the surface free energy of the materials. As the steps of crosslinking and GO filling proceed, there are less reactive moieties available at the interface, since covalent bonds stabilize the copolymer, and GO sheets enhance the strength of the macromolecular network by additional H bonding. Additionally, GO might impact the measurements due to its intrinsic quenching ability, as the fluorescence is diminished with the GO ratio to copolymer increase. Still, this composite copolymer can be a good candidate for applications where antifouling properties are required.

## 3. Materials and Methods

### 3.1. Materials

Firstly, precursors for the copolymer series synthesis, graphene oxide, crab shell-derived medium molecular weight chitosan, genipin (≥98%, High Performance Liquid Chromatography (HPLC) grade) and acetic acid (≥99.7%), N-(3-dimethylaminopropyl)-N′-ethylcarbodiimide hydrochloride—EDC (≥98%), N-hydroxysuccinimide—NHS (98%), ethanol (99.8%), methanol (99.8%), palmitic acid and stearic acid (>95%) were purchased from Sigma-Aldrich, St. Louis, MI, USA. All materials were used without additional purification. The water used in this work was double distilled water. Enzyme degradation experiments involved lysozyme (from chicken egg white lyophilized powder, protein ≥90%, ≥40,000 units/mg protein), sodium azide (≥99.5%), calcium chloride (≥97%), and Tris-HCl, ethylenediaminetetraacetic acid (≥99%) purchased from Sigma-Aldrich St. Louis, MI, USA, and used without additional purification. Phosphate buffer saline (tablets, pH 7.4, Sigma Aldrich, St. Louis, MI, USA) was used to prepare the aqueous (in double distilled water) solution.

### 3.2. Chitosan Modification

Fatty acid modified chitosan was synthesized by the EDC-NHS coupling mechanism, using two conventional methods [5,6]. Medium molecular weight chitosan was solubilized in aqueous solution of acetic acid (pH 4) overnight at 40° C to form a diluted solution (0.5 wt.%). The fatty acid mixture consisted of a molar ratio between palmitic and lauric acid of 1:1. The fatty acid carboxyl to chitosan amine ratio was fixed to 0.5 M/mol of D-glucosamine unit. A 1% solution of fatty acids was obtained in 1:2 *v*/*v* of methanol and ethanol at room temperature, by stirring for 1 h. The EDC and NHS to fatty acid molar ratio was 1:1. EDC was added to the FA solution and stirred for 1 h under normal conditions in order to form O-acylisourea active ester intermediate. Then, NHS addition stabilized the system by enabling carbodiimide to couple NHS to the carboxyl group, forming an amine reactive ester. The reaction continued for 24 h under vigorous stirring conditions at 90 °C. The product was dialyzed for 48 h against double distilled water. Modified chitosan (CHT-g-FA) was freeze-dried (frozen at −70 °C, dried at −50°), and the residual unreacted fatty acids were removed by ethanol rinsing.

### 3.3. CHT-g-FA/GO Composites Synthesis

Unlike CHT, CHT-g-FA is water soluble regardless of the solution’s pH; the CHT-g-FA batch was solubilized in double distilled water (2 wt.% at 40 °C). CHT-g-FA was crosslinked with genipin (Gp) at room temperature (0.5 wt.%). Crosslinked formulations were enhanced with graphene oxide in 0.5 and 2 wt.% reported to the total macromolecular chain mass. Graphene oxide was dispersed in double distilled water for 1 h and corresponding volumes were added to the aqueous CHT-g-FA solution. Then, the formulations were placed in Petri dishes and left undisturbed to allow for water evaporation. The batch of modified CHT materials is catalogued in Table 2 below.

### 3.4. Fourier-Transform Infrared Spectrometry (FTIR)

A Vertex 70 Bruker FTIR spectrometer with an attenuated total reflectance (ATR) accessory was used to perform the FTIR measurements on film specimens. In the ATR-FTIR mode, 32 scans were registered for each sample at room temperature with a resolution of 4 cm^−1^ in the 600–4000 cm^−1^ wavenumber range.

### 3.5. Dynamic Light Scattering (DLS)

A Dynamic Light Scattering (Zetasizer Nano ZS, Malvern Instrument) device equipped with a He/Ne laser was used to assess the hydrodynamic diameter (d), diffusion coefficient (D), polydispersity (PdI), and zeta potential (ζ). The electrophoretic mobility experiments (converted to zeta potentials using the Helmholtz–Smoluchowski equation) were carried out at a scattering angle of 13°, while the size measurements were carried out at a scattering angle of 173°. DLS measurements were performed on aqueous samples with concentrations of 0.1 wt.% using plastic capillary cells with electrodes at either end (Malvern Instruments). The samples were equilibrated in the instrument for 120 s at 25 °C, then 15 cycles were conducted for each specimen, the tests were performed in triplicate, and the data were provided with standard deviation.

### 3.6. Contact Angle Measurements

A Kruss DSA100S apparatus, equipped with a CF03 digital camera, was used to study the wetting properties of all crosslinked scaffolds as well as the surface free energy of the materials. Using the sessile drop method, the contact angle was determined 10 s after the droplet was deposited. At room temperature, at least three measurements were taken in various areas on the sample. The averaged values were calculated and reported using the Young Laplace equation. Surface free energy (SFE) is a term that is used to characterize a solid and is mathematically equivalent to liquid surface tension. The SFE of a material dictates how it reacts when it comes into contact with other materials. High SFE materials are unstable from a thermodynamic standpoint, and contact must be minimized.

### 3.7. Nanoindentation

Mechanical properties at the surface were determined through nanoindentation tests using a Nano Indenter ^®^ G200 (KLA Instruments) equipped with CSM option and a DCM II head. The method used provides Young’s modulus (E) and hardness (H) as a continuous function of indentation depth [54]. Ten indentations (500 nm depth) were performed on each sample using a Berkovich diamond tip with a radius of 20 nm. To avoid any interference between each indentation, at least 25 μm distance was maintained between them. The results are expressed as mean (*n* = 10) ± standard deviation. The 10% rule of thumb, which says that for free substrate influence results, the indentation depth should not exceed 10% of film thickness, was applied [55].

### 3.8. Scanning Electron Microscopy (SEM)

SEM experiments were carried out on a Versa Three-Dimensional (3-D) Dual Beam Field Emission Scanning Electron Microscope (FEI Company), under high vacuum (<3 × 10^−4^ Pa). The specimens were sputter-coated with a thin gold coating, and their morphology was examined using a 5, 10 and 20 kV field emission gun. An Everhart–Thornley Detector (ETD) for Secondary Electrons signal detection was used to examine the complex topography of the analyzed surfaces.

### 3.9. Swelling Degree

The water uptake of the modified chitosan batch materials was evaluated by calculating the percentage of weight variation after sample immersion in PBS solution (pH 7.4, 37° C). Dry pre-weighed (w_d_) disc-shaped specimens were hydrated for pre-determined extents (t_j_) then removed from the incubation medium and weighed after blotting the excess solution (w_tj_). Swelling capacity [%] was calculated from Equation (1). The swelling profile (Figure 7) depicts the averaged values of triplicate measurements.

Δs [%] = 100 × (w_tj_ − w_d_)/w_d_ (1)

Δs is the swelling degree and w_tj_ is the swollen mass of the specimen.

### 3.10. Degradability Assessment

The modified chitosan degradation was assessed in two media: phosphate buffer saline (PBS) at pH 7.4 and 37° C, as well as lysozyme rich solution (10 µg/mL). Enzyme solution was obtained in 10 mM Tris-HCl, 10 mM NaCl, 10 mM EDTA and 10 mM NaN_3_ aqueous solution (all purchased from Sigma-Aldrich, St. Louis, MI, USA). Degradation behavior was monitored for up to 8 weeks. Samples were weighed before (w_i_) and after (w_f_) in vitro degradation in order to determine the weight loss degree (Δw) according to Equation (2). The curves depict the averaged values of triplicate measurements.

Δw [%] = 100 × (w_i_ − w_f_)/w_i_ (2)

### 3.11. In Vitro Biocompatibility Assessment

#### 3.11.1. Cell Seeding

L929 fibroblast cell line (85011425 from ECACC) was cultured in Dulbecco’s Modified Eagle Medium (DMEM from LonzaBioWhittaker, MD, USA) containing 4.5 g/L glucose and L-Glutamine (LonzaBioWhittaker, Verviers, Belgium), supplemented with 10% heat-inactivated fetal bovine serum (FBS, Thermo Fisher Scientific, Waltham, MA, USA), 2% 4 mM L-glutamine (Sigma, St. Louis, MI, USA) and 100 U/100 mg mL penicillin/streptomycin (Pen/Strep, from LonzaBioWhittaker, MD, USA). The fibroblasts thus obtained were seeded in a 96-well plate at a density of 20,000 cells/well to perform the cytotoxicity tests, according to ISO standard 10993-5 guidelines.

#### 3.11.2. Cytotoxicity Investigations

Samples of interest were added to a Falcon tube (Corning, NY, USA) containing cell culture medium (DMEM), to reach a final concentration of 4 wt.%. Post incubation at 37 °C, part of the medium (1 mL) was collected at fixed time points (2, 4, 24, 72 h, 7 and 14 days). The same volume of fresh DMEM medium was added to the Falcon tube and the incubation was allowed to continue. Supernatants thus collected were subsequently used for the cytotoxicity investigations and stored at −20 °C up to 4 weeks before use.

L929 fibroblast cells at a confluency close to 80–90% were stimulated with the collected supernatants and incubated at 37 °C (5% CO_2_). After a 24 h incubation, the cell viability was determined using a 3-[4,5-dimethylthiazole-2-yl]-2,5-diphenyltetrazolium bromide (MTT, from Sigma-Aldrich, St. Louis, MI, USA) assay, following the conditions recommended by the manufacturer. Briefly, the stimulated cells were incubated at 37 °C in a culture medium containing 0.5 mg/mL MTT, 100 µL/well. After 3 h of incubation at 37 °C, the blue MTT–formazan product was first extracted with 100 µL/well lysis buffer (20% sodium dodecyl sulphate, SDS 98.5% from Sigma-Aldrich, St. Louis, MI, USA, 50% dimethylformaldehyde, DMF 99.9% from Sigma-Aldrichm, St. Louis, MI, USA, 0.4% acetic acid from Chemical Company and 0.04 N hydrochloric acid) for solvation of formazan crystals, and after another 48 h at room temperature extraction, the absorbance of the formazan solution was read with an ELISA Multiskan FC reader from ThermoScientific at 570 nm, using SkanIt RE 4.1 software.

The MTT reduction by healthy cells and the relative cell viability was calculated as the ratio between the stimulated cells samples’ absorbance (Abs) and control absorbance measured for unstimulated cells:

$$Relative\;cell\;viability\;\left(\%\right)=\frac{Abs_{sample}}{Abs_{control}}$$

In parallel, treated cells with Triton-X 0.2% (1:10 *v*/*v* to fresh cell medium, from Sigma-Aldrich, St. Louis, MI, USA) were used as positive controls, while negative controls were obtained by the incubation of healthy cells in freshly prepared culture medium and were considered as 100% in cell viability.

A Lactate Dehydrogenase Assay (LDH) was also applied as a complementary method to investigate cytotoxicity. LDH is an enzyme present when a cell membrane becomes damaged, thus making it a widely used to assess cytotoxicity assays. The protocol is based on an enzymatic coupling reaction: LDH released from the cell oxidizes lactate to generate NADH, which through reaction with a water-soluble tetrazolium salt produces a yellow color. The intensity of the generated color correlates directly with the number of lyzed cells. In the present study, the manufacturer’s instructions were followed for this assay, with slight modifications for the use of a 384-well plate. Briefly, 12.5 µL from the collected supernatants was added to a 384-well plate together with similar volumes of LDH aliquoted solution and stop solution (LDH Kit, from Promega Co, WI, USA). After a 30 min at room temperature, the color intensity was read at OD 570 nm using a multiplate reader, Multiskan Fc, Thermo Scientific.

#### 3.11.3. Expression of IL -6 Inflammatory Marker

The recombinant mouse interleukin 6 (IL6) expression of the above-mentioned stimulated fibroblasts with collected supernatants was recorded using DuoSet^®^ ELISA DEVELOPMENT SYSTEM kits (R&D Systems, MN, USA) as recommended by the manufacturer. Briefly, an ELISA sandwich technique was applied, where the 384-well plates for IL6 were initially coated with 2μg/mL Rat Anti-Mouse IL-6 Capture Antibody and left over overnight at room temperature. The next day, the coated plate was washed (three times) with Wash Buffer (0.05% Tween 20 in PBS, pH 7.2–7.4), and left to block with 1% BSA in PBS (pH 7.2–7.4) for 2 h at room temperature. After another washing step, the samples and seven points of Recombinant Mouse IL-6 Standard (1000 pg/mL high point) using 2-fold serial dilutions in Reagent Diluent (1% BSA in PBS; pH 7.2–7.4) was added. After 2 h of incubation at room temperature and a washing step, 150 ng/mL specific Biotinylated Goat Anti-Mouse IL-6 Detection Antibody was added. Following 2 h of incubation at room temperature and a washing step, Streptavidin conjugated to horseradish-peroxidase was added, and the plate was incubated for 20 min at dark. Subsequently, unbounded Streptavidin-HRP was washed and the colorimetric substrate 3,3′,5,5;-TetraMethylBenzidine (TMB) was added to each well, left to react for 20 min at dark and finally stopped using H_2_SO_4_ 1 N. The optical density was determined immediately at 450 nm using SkanIt RE 4.1 software equipped to a microplate reader, Thermo Scientific Multiskan FC, ThermoScientific.

Levels of proinflammatory cytokine IL-6 were calculated using regression equation values of a plot of four parameters logistic curve by placing each standard concentration values on the Y axis and respective optical density values on the X axis.

#### 3.11.4. Protein Adsorption Investigations

The protein adsorption on various chitosan derivative surfaces was investigated via fluorescent microscope imaging with the help of a model fluorescently labelled protein such as bovine serum albumin (BSA) marked with a fluorescein isothiocyanate conjugate (FITC) moiety. Four modified chitosan discs of similar dimensions were incubated in a 1 mg/mL BSA-FITC solution in phosphate buffer at room temperature at dark. After an overnight incubation, the samples were washed twice for 10 min with freshly prepared phosphate buffer to remove the excess of adsorbed protein. In parallel, unlabeled reference samples were prepared by sample incubation in phosphate buffer as above, in the absence of the protein.

A Nikon Eclipse Ti (Nikon) inverted microscope equipped with a Nikon DS-Qi2 monochrome camera (Nikon) and a green fluorescent protein (GFPHQ, Ex = 455–485 nm) filter (Nikon) was employed to record phase contrast and fluorescence images of the treated samples, respectively. To obtain a better visualization, the samples were mounted between glass slides using a drop of aqueous medium to maintain the sample hydrated. One phase contrast image was taken for each sample at a 4x magnification, 10 ms exposure, and 4 different images were taken at a 20x magnification under fluorescence light, 25 ms exposure, using the GFPHQ filter. The fluorescence intensity values were recorded as the mean value of four different measurements. The images and data were processed using the Micro-Manager and Fiji ImageJsoftware.

## 5. Conclusions

In this study, four formulations derived from fatty acid modified chitosan were synthesized and characterized. First a chitosan-fatty acids copolymer was synthesized by covalent coupling through a EDC-NHS reaction mechanism. The amphiphilic macromolecule was further crosslinked with genipin and reinforced with different weight percentages of graphene oxide (0.5% and 2%).

Copolymerization, crosslinking and compositing were intended as a cascading ap-proach to modulate polymer features. These procedures gradually modified the structural and morphological features of the material, as confirmed by FTIR, DLS and SEM. These heterogeneities could favor cell adhesion and further stimulate their proliferation. As a result of the coupling reaction, surface roughness was increased and wettability was improved as hydrophobic FA chains organized towards the inner areas. In the case of contact angle studies, the system containing 0.5% GO exhibited the highest hydrophilicity, while the character of the 2% GO reinforced system was less hydrophilic because GO sheets are inclined to set apart into compact structures. The swelling studies presented in this study confirm that as the heterogeneity in composite networks increases, the water affinity of multi-component systems increases. PBS and lysozyme degradation assays indicated that GO-copolymer composites demonstrated the most stable behavior. GO compositing also enhanced the nanomechanical features of the samples.

According to the biological tests, it was observed that the viability was favorable for all materials at each measurement moment, as the indirect viability quantified by the MTT test exceeded 80%. In addition, genipin crosslinked materials proved to be superior to the control sample, and GO composites, 2 wt.% reinforced in particular, led to the fewest cytotoxicity variations over time. Furthermore, the GO composite, due to the lower free surface energy, retains less BSA, which is an indication of a possible antifouling behavior that would need to be carefully addressed in a future study.

To sum up, we have designed through an accessible method, materials used to fabricate films which are anisotropic in nature. Both microscopic and molecular anisotropy, such as topography and molecular-level interactions, durotactic gradients and phase distributions were examined. The formulations obtained may improve cell adhesion and proliferation and could potentially be used in various biomedical applications, including—scaffolding as well as coatings with antifouling or cell-friendlier features.

## Figures and Tables

**Table 1:** Hydrodynamic features of precursor polymer and newly developed systems.

Sample	d (nm)	PdI	ζ (mV)	D (μm^2^ s^−1^)
CHT	751.6 ± 52.6	0.741 ± 0.048	+31.6 ± 0.854	0.711
CHT-g-FA	536.0 ± 7.35	0.488 ± 0.089	+35.3 ± 0.6	0.923
CHT-g-FA/Gp	756.9 ± 57.93	0.713 ± 0.074	+51.4 ± 1.58	0.703
CHT-g-FA/Gp/GO05	721.3 ± 38.95	0.789 ± 0.035	+33.4 ± 0.32	0.645
CHT-g-FA/Gp/GO2	666.1 ± 26.55	0.572 ± 0.068	+33.4 ± 0.36	0.759

**Table 2:** Modified CHT-based materials. Gp and GO percentages concern the polymer mass.

Sample Name	Chitosan	Gp [wt.%]	GO [wt.%]
CHT-g-FA	☑	0	0
CHT-g-FA/Gp	☑	0.5	0
CHT-g-FA/Gp/GO05	☑	0.5	0.5
CHT-g-FA/Gp/GO2	☑	0.5	2

## Data Availability

Not applicable.

## Supplementary Materials

The following supporting information can be downloaded at: https://www.mdpi.com/article/10.3390/ijms23105336/s1.

## References

[1] Zhao X., Hu D.A., Wu D., He F., Wang H., Huang L., Shi D., Liu Q., Ni N., Pakvasa M. (2021). Applications of Biocompatible Scaffold Materials in Stem Cell-Based Cartilage Tissue Engineering. Front. Bioeng. Biotechnol..

[2] Farzan A., Borandeh S., Ezazi N.Z., Lipponen S., Santos H.A., Seppälä J. (2020). 3D scaffolding of fast photocurable polyurethane for soft tissue engineering by stereolithography: Influence of materials and geometry on growth of fibroblast cells. Eur. Polym. J..

[3] Palma P.J., Ramos J.C., Martins J.B., Diogenes A., Figueiredo M.H., Ferreira P., Viegas C., Santos J.M. (2017). Histologic evaluation of regenerative endodontic procedures with the use of chitosan scaffolds in immature dog teeth with apical periodontitis. J. Endod..

[4] Dhandayuthapani B., Yoshida Y., Maekawa T., Kumar D.S. (2011). Polymeric scaffolds in tissue engineering application: A review. Int. J. Polym. Sci..

[5] Sharma D., Singh J. (2017). Synthesis and characterization of fatty acid grafted chitosan polymer and their nanomicelles for nonviral gene delivery applications. Bioconj. Chem..

[6] El Fray M., Niemczyk A., Pabin-Szafko B. (2012). Chemical modification of chitosan with fatty acids. Prog. Chem. Appl. Chitin. Deriv..

[7] Niemczyk A., Goszczyńska A., Gołda-Cępa M., Kotarba A., Sobolewski P., El Fray M. (2019). Biofunctional catheter coatings based on chitosan-fatty acids derivatives. Carbohydr. Polym..

[8] Yu Y., Xu S., Li S., Pan H. (2021). Genipin-cross-linked hydrogels based on biomaterials for drug delivery: A review. Biomater. Sci..

[9] Adorinni S., Rozhin P., Marchesan S. (2021). Smart Hydrogels Meet Carbon Nanomaterials for New Frontiers in Medicine. Biomedicines.

[10] Jamilpour N., Fereidoon A., Rouhi G. (2011). The effects of replacing collagen fibers with carbon nanotubes on the rate of bone remodeling process. J. Biomed. Nanotechnol..

[11] Mansouri N., Al-Sarawi S., Losic D., Mazumdar J., Clark J., Gronthos S., Doig R. (2021). Biodegradable and Biocompatible Graphene-Based Scaffolds for Functional Neural Tissue Engineering: A Strategy Approach Using Dental Pulp Stem Cells and Biomaterials. Biotechnol. Bioeng..

[12] Driscoll J., Moirangthem A., Yan I.K., Patel T. (2021). Fabrication and characterization of a biomaterial based on extracellular-vesicle functionalized graphene oxide. Front. Bioeng. Biotechnol..

[13] Phan L.M.T., Vo T.A.T., Hoang T.X., Cho S. (2021). Graphene integrated hydrogels based biomaterials in photothermal biomedicine. Nanomaterials.

[14] Vlasceanu G.M., Șelaru A., Dinescu S., Balta C., Herman H., Gharbia S., Hermenean A., Ionita M., Costache M. (2020). Comprehensive Appraisal of Graphene–Oxide Ratio in Porous Biopolymer Hybrids Targeting Bone-Tissue Regeneration. Nanomaterials.

[15] Vlasceanu G.M., Crica L.E., Pandele A.M., Ionita M. (2020). Graphene oxide reinforcing genipin crosslinked chitosan-gelatin blend films. Coatings.

[16] Thotakura N., Dadarwal M., Kumar R., Singh B., Sharma G., Kumar P., Katare O.P., Raza K. (2017). Chitosan-palmitic acid based polymeric micelles as promising carrier for circumventing pharmacokinetic and drug delivery concerns of tamoxifen. Int. J. Biol. Macromol..

[17] Tran T.-Q.-M., Hsieh M.-F., Chang K.-L., Pho Q.-H., Nguyen V.-C., Cheng C.-Y., Huang C.-M. (2016). Bactericidal effect of lauric acid-loaded PCL-PEG-PCL nano-sized micelles on skin commensal Propionibacterium acnes. Polymers.

[18] Karim N., Shishir M.R.I., Rashwan A.K., Ke H., Chen W. (2021). Suppression of palmitic acid-induced hepatic oxidative injury by neohesperidin-loaded pectin-chitosan decorated nanoliposomes. Int. J. Biol. Macromol..

[19] Wang N., Yu H., Song Q., Mao P., Li K., Bao G. (2021). Sesamol-loaded stearic acid-chitosan nanomicelles mitigate the oxidative stress-stimulated apoptosis and induction of pro-inflammatory cytokines in motor neuronal of the spinal cord through NF-ĸB signaling pathway. Int. J. Biol. Macromol..

[20] Hu X., Fatima S., Chen M., Xu K., Huang C., Gong R.-H., Su T., Wong H.L.X., Bian Z., Kwan H.Y. (2021). Toll-like receptor 4 is a master regulator for colorectal cancer growth under high-fat diet by programming cancer metabolism. Cell Death Dis..

[21] Sun Y., Nan D., Jin H., Qu X. (2020). Recent advances of injectable hydrogels for drug delivery and tissue engineering applications. Polym. Test..

[22] Vlăsceanu G.M., Iovu H., Ioniţă M. (2019). Graphene inks for the 3D printing of cell culture scaffolds and related molecular arrays. Compos. B. Eng..

[23] Cernencu A.I., Lungu A., Stancu I.-C., Serafim A., Heggset E., Syverud K., Iovu H. (2019). Bioinspired 3D printable pectin-nanocellulose ink formulations. Carbohydr. Polym..

[24] Zhang L., Li X., Shi C., Ran G., Peng Y., Zeng S., He Y. (2021). Biocompatibility and Angiogenic Effect of Chitosan/Graphene Oxide Hydrogel Scaffolds on EPCs. Stem Cells Int..

[25] Bhattacharjee P., Ahearne M. (2021). Significance of Crosslinking Approaches in the Development of Next Generation Hydrogels for Corneal Tissue Engineering. Pharmaceutics.

[26] Upadhyay R. (2017). Use of polysaccharide hydrogels in drug delivery and tissue engineering. Adv. Tissue Eng. Regen. Med. Open Access.

[27] Plucinski A., Lyu Z., Schmidt B.V. (2021). Polysaccharide nanoparticles: From fabrication to applications. J. Mater. Chem. B.

[28] Lamptey R.N.L., Gothwal A., Trivedi R., Arora S., Singh J. (2022). Synthesis and Characterization of Fatty Acid Grafted Chitosan Polymeric Micelles for Improved Gene Delivery of VGF to the Brain through Intranasal Route. Biomedicines.

[29] Balan V., Mihai C.-T., Cojocaru F.-D., Uritu C.-M., Dodi G., Botezat D., Gardikiotis I. (2019). Vibrational spectroscopy fingerprinting in medicine: From molecular to clinical practice. Materials.

[30] Dimzon I.K.D., Knepper T.P. (2015). Degree of deacetylation of chitosan by infrared spectroscopy and partial least squares. Int. J. Biol. Macromol..

[31] Pereira K.A.A., Osório L.R., Silva M.P., Sousa K.S., Silva Filho E.C.d. (2014). Chemical modification of chitosan in the absence of solvent for diclofenac sodium removal: pH and kinetics studies. Mater. Res..

[32] Wiercigroch E., Szafraniec E., Czamara K., Pacia M.Z., Majzner K., Kochan K., Kaczor A., Baranska M., Malek K. (2017). Raman and infrared spectroscopy of carbohydrates: A review. Spectrochim. Acta A Mol. Biomol. Spectrosc..

[33] Hayashi K., Mitsuyoshi Y., Kamei T., Shimanouchi T., Suga K., Okamoto Y., Nakamura H., Umakoshi H. (2018). Design of Pyrene–Fatty Acid Conjugates for Real-Time Monitoring of Drug Delivery and Controllability of Drug Release. ACS Omega.

[34] Silva M.C., Andrade C.T. (2009). Evaluating conditions for the formation of chitosan/gelatin microparticles. Polimeros.

[35] Wang X., Tang R., Zhang Y., Yu Z., Qi C. (2016). Preparation of a novel chitosan based biopolymer dye and application in wood dyeing. Polymers.

[36] Klein M.P., Hackenhaar C.R., Lorenzoni A.S., Rodrigues R.C., Costa T.M., Ninow J.L., Hertz P.F. (2016). Chitosan crosslinked with genipin as support matrix for application in food process: Support characterization and β-d-galactosidase immobilization. Carbohydr. Polym..

[37] Pandele A.M., Andronescu C., Vasile E., Radu I.C., Stanescu P., Iovu H. (2017). Non-covalent functionalization of GO for improved mechanical performances of pectin composite films. Compos.-A Appl. Sci. Manuf..

[38] Olaret E., Ghitman J., Iovu H., Serafim A., Stancu I.C. (2020). Coatings based on mucin-tannic acid assembled multilayers. Influence of pH. Polym. Adv. Technol..

[39] Ghitman J., Biru E.I., Cojocaru E., Pircalabioru G.G., Vasile E., Iovu H. (2021). Design of new bioinspired GO-COOH decorated alginate/gelatin hybrid scaffolds with nanofibrous architecture: Structural, mechanical and biological investigations. RSC Adv..

[40] Danaei M., Dehghankhold M., Ataei S., Hasanzadeh Davarani F., Javanmard R., Dokhani A., Khorasani S., Mozafari M. (2018). Impact of particle size and polydispersity index on the clinical applications of lipidic nanocarrier systems. Pharmaceutics.

[41] Akopova T.A., Demina T.S., Khavpachev M.A., Popyrina T.N., Grachev A.V., Ivanov P.L., Zelenetskii A.N. (2021). Hydrophobic Modification of Chitosan via Reactive Solvent-Free Extrusion. Polymers.

[42] Mielan B., Sousa D.M., Krok-Borkowicz M., Eloy P., Dupont C., Lamghari M., Pamuła E. (2021). Polymeric Microspheres/Cells/Extracellular Matrix Constructs Produced by Auto-Assembly for Bone Modular Tissue Engineering. Int. J. Mol. Sci..

[43] Cojocaru E., Ghitman J., Biru E.I., Pircalabioru G.G., Vasile E., Iovu H. (2021). Synthesis and Characterization of Electrospun Composite Scaffolds Based on Chitosan-Carboxylated Graphene Oxide with Potential Biomedical Applications. Materials.

[44] Komartin R.S., Balanuca B., Necolau M.I., Cojocaru A., Stan R. (2021). Composite Materials from Renewable Resources as Sustainable Corrosion Protection Coatings. Polymers.

[45] Zonderland J., Moroni L. (2020). Steering cell behavior through mechanobiology in 3D: A regenerative medicine perspective. Biomaterials.

[46] Şelaru A., Drăgușin D.-M., Olăreț E., Serafim A., Steinmüller-Nethl D., Vasile E., Iovu H., Stancu I.-C., Costache M., Dinescu S. (2019). Fabrication and biocompatibility evaluation of nanodiamonds-gelatin electrospun materials designed for prospective tissue regeneration applications. Materials.

[47] Gupta T.K., Singh B.P., Tripathi R.K., Dhakate S.R., Singh V.N., Panwar O., Mathur R.B. (2015). Superior nano-mechanical properties of reduced graphene oxide reinforced polyurethane composites. RSC Adv..

[48] Fan H., Wang L., Zhao K., Li N., Shi Z., Ge Z., Jin Z. (2010). Fabrication, mechanical properties, and biocompatibility of graphene-reinforced chitosan composites. Biomacromolecules.

[49] Papageorgiou D.G., Kinloch I.A., Young R.J. (2017). Mechanical properties of graphene and graphene-based nanocomposites. Prog. Mater. Sci..

[50] Zhang D., Yang S., Chen Y., Liu S., Zhao H., Gu J. (2018). 60Co γ-ray irradiation crosslinking of chitosan/graphene oxide composite film: Swelling, thermal stability, mechanical, and antibacterial properties. Polymers.

[51] Johnson B.Z., Stevenson A.W., Prêle C.M., Fear M.W., Wood F.M. (2020). The role of IL-6 in skin fibrosis and cutaneous wound healing. Biomedicines.

[52] Cicuéndez M., Casarrubios L., Barroca N., Silva D., Feito M.J., Diez-Orejas R., Marques P.A., Portolés M.T. (2021). Benefits in the Macrophage Response Due to Graphene Oxide Reduction by Thermal Treatment. Int. J. Mol. Sci..

[53] Bellet P., Gasparotto M., Pressi S., Fortunato A., Scapin G., Mba M., Menna E., Filippini F. (2021). Graphene-based scaffolds for regenerative medicine. Nanomaterials.

[54] Hay J., Agee P., Herbert E. (2010). Continuous stiffness measurement during instrumented indentation testing. Exp. Tech..

[55] Fischer-Cripps A., Johnson K. (2002). Introduction to Contact Mechanics. Mechanical Engineering Series. Appl. Mech. Rev..

