# Supplementary material for: Chitosan-Based Materials Featuring Multiscale Anisotropy for Wider Tissue Engineering Applications
Source: Int J Mol Sci. 2022 May 10;23(10):5336. doi: 10.3390/ijms23105336 (PMC9140409; doi:10.3390/ijms23105336)
Supplement: Supplementary file 1 [file ijms-23-05336-s001.zip › ijms-1703920-supplementary.pdf]

I. Topography assessment

The topography of the specimens was illustrated by processing the scanning electron microscopy (SEM) images of the samples (depicted in the manuscript body) in ImageJ software (1.53 k, Java 1.8.0_331 64-bit). In brief, each SEM image was loaded, the scale bar was set in the software manually (10 μm for the 5000 × magnification, 5 μm for the 10000 × magnification and 3 μm for the 5000 × magnification); a square shaped area of each microscopy image was selected and "Surface plot" facility was activated from "Analysis" menu. The resulted topographies are depicted in Figure S1 below.

The features of the sample topographies are in agreement with the SEM observations discussed in the manuscript. The surface roughness is depicted as a function of contrast, whereby the lighter areas are associated to the most convex and irregular domains within the captured plane. The control, CHT-g-FA, exhibits a rough surface, with specific protuberances due to the dome-shaped structuration of the material. Upon genipin crosslinking, smoother surfaces were formed due to the constrictions covalent bonding the copolymer chains induced. Graphene oxide compositing favoured the formation of rough surfaces again, with a maximum achieved for the 2 wt% GO additivation.

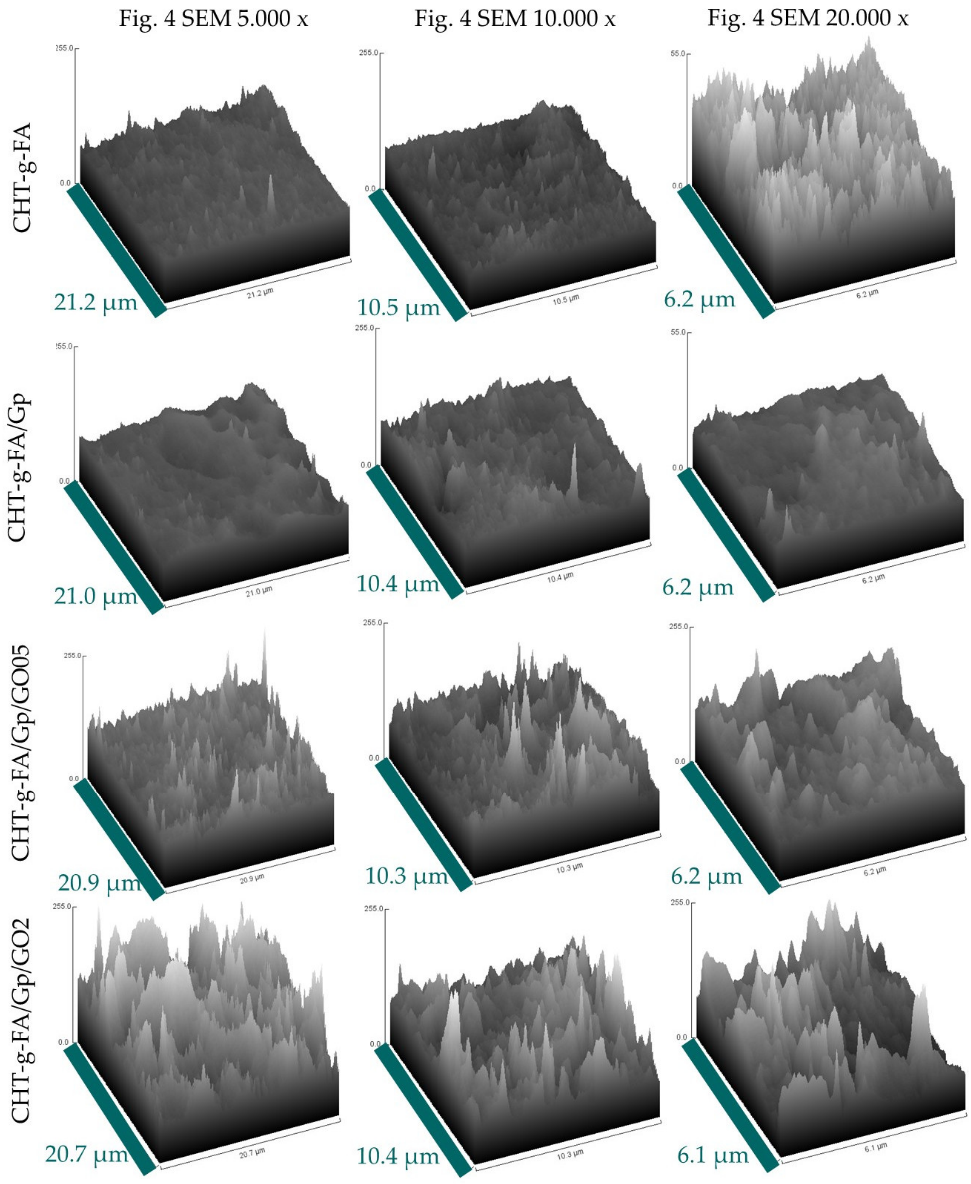

Figure S1. Topography plots of the synthesized materials based on the SEM images.

II. Biological assessment. Statistical analysis

GraphPad Prism 9 (USA) for Windows was used to statistically analyse the results of cytotoxicity investigations (MTT – Table S1 and LDH assays – Table S2) and expression of IL-6 inflammatory marker (Table S3). All presented quantitative results plotted in the main body of the manuscript are the mean ± standard deviation (SD) of n = 3 experiments; the calculated p-values are accounted below in order to preserve the clarity of the figure. The result were compared using one-way ANOVA and Bonferroni post-test (* - $p<0.05$, ** - $p<0.01$, *** - $p<0.001$, ns – not significant. p-values < 0.05 were considered to be statistically significant.

Table S1. Tabulation of the p-values calculated for the MTT assay.

MTT/ p-values	CHT-g-FA	CHT-g-FA/Gp	CHT-g-FA/Gp/GO05	CHT-g-FA/Gp/GO2
CHT-g-FA		*	*	ns
CHT-g-FA/Gp	*		*	**
CHT-g-FA/Gp/GO05	*	*		ns
CHT-g-FA/Gp/GO2	ns	**	ns	

Table S2. Tabulation of the p-values calculated for the LDH assay

LDH/ p-values	CHT-g-FA	CHT-g-FA/Gp	CHT-g-FA/Gp/GO05	CHT-g-FA/Gp/GO2
CHT-g-FA		ns	**	***
CHT-g-FA/Gp	ns		**	***
CHT-g-FA/Gp/GO05	**	**		**
CHT-g-FA/Gp/GO2	***	***	**	

Table S3. Tabulation of the p-values calculated for the IL-6 expression

IL-6/ p-values	CHT-g-FA	CHT-g-FA/Gp	CHT-g-FA/Gp/GO05	CHT-g-FA/Gp/GO2
CHT-g-FA		ns	***	*
CHT-g-FA/Gp	ns		***	ns
CHT-g-FA/Gp/GO05	***	***		ns
CHT-g-FA/Gp/GO2	*	ns	ns	
